# Long-Term Outcomes of Bioprosthetic and Mechanical Valve Replacement for Patients Aged between 50 and 70 Years

**DOI:** 10.31083/j.rcm2409253

**Published:** 2023-09-18

**Authors:** Wei Zhao, Zhongli Chen, Sipeng Chen, Junzhe Du, Heng Zhang, Yan Zhao, Li He, Wei Feng, Hansong Sun, Zhe Zheng

**Affiliations:** ^1^National Clinical Research Center of Cardiovascular Diseases, State Key Laboratory of Cardiovascular Disease, Fuwai Hospital, National Center for Cardiovascular Diseases, Chinese Academy of Medical Sciences and Peking Union Medical College, 100037 Beijing, China; ^2^Department of Cardiovascular Surgery, Fuwai Hospital, National Center for Cardiovascular Diseases, Chinese Academy of Medical Sciences and Peking Union Medical College, 100037 Beijing, China; ^3^State Key Laboratory of Cardiovascular Disease, Cardiac Arrhythmia Center, Fuwai Hospital, National Center for Cardiovascular Disease, Chinese Academy of Medical Sciences and Peking Union Medical College, 100037 Beijing, China; ^4^Information Center, Fuwai Hospital, National Center for Cardiovascular Diseases, Chinese Academy of Medical Sciences and Peking Union Medical College, 100037 Beijing, China; ^5^Department of Cardiothoracic Surgery, Shenzhen Children’s Hospital, 518026 Shenzhen, Guangdong, China

**Keywords:** valvular heart disease, bioprosthetic valve, mechanical valve, valve replacement

## Abstract

**Background:**

The choice between bioprosthetic and mechanical valves for aortic valve replacement (AVR) and mitral valve replacement (MVR) among patients aged 50–70 years is controversial. We compared the long-term outcomes of patients using bioprosthetic or mechanical valves to provide clinical evidence for valve selection.

**Methods:**

From 2002 to 2007, patients aged 50–70 years who underwent isolated AVR or MVR at the Fuwai Hospital were enrolled. After inverse probability-weighted (IPW) propensity balancing, we evaluated long-term mortality, stroke, and bleeding events between patients receiving mechanical and biological prostheses for MVR or AVR.

**Results:**

A total of 1639 patients were included in the study, including 1181 patients undergoing MVR (median follow-up: 11.6 years) and 458 patients undergoing AVR (median follow-up: 11.4 years). After IPW adjustment, there was no significant difference in long-term mortality and stroke rate between patients using bioprosthetic and mechanical valves for MVR [mortality: log-rank *p = *0.802; stroke: log-rank *p = *0.983] and AVR [mortality: log-rank *p = *0.815; stroke: log-rank *p = *0.537]. Landmark analysis at 12.5 years yielded significantly lower mortality in the patients receiving mechanical valves compared with bioprosthetic valves in the MVR cohort (*p = *0.028). Patients receiving mechanical aortic valves displayed an increased risk of bleeding compared with those who received bioprosthetic aortic valves [Hazard Ratio (95% Confidence interval): 2.51 (1.06–5.93) *p = *0.036].

**Conclusions:**

For patients aged 50–70, there was no significant difference in overall long-term mortality between mechanical and bioprosthetic valve recipients. Patients receiving mechanical valves for MVR displayed lower mortality after 12.5 years follow-up. For AVR, bioprosthetic valves were associated with a lower risk of bleeding.

## 1. Introduction

Valve replacement has been proven to be an effective treatment for improving the prognosis of patients with severe valvular disease [1, 2]. However, for patients undergoing valve replacement, the choice between biological and mechanical valves is always challenging because the outcome can be affected by the tradeoff between prosthesis durability, hemodynamics, and risk of hemorrhage thromboembolism [3, 4].

The age cut-off for prosthesis selection has been addressed in various international guidelines, but is inconsistent for those patients between 50 and 70 years [5, 6, 7, 8]. Current European Society of Cardiology (ESC)/European Association for Cardio-Thoracic Surgery (EACTS) guidelines recommend mechanical valves in patients younger than 60 years old and 65 years old for aortic valve replacement (AVR) and mitral valve replacement (MVR), respectively, and bioprosthetic valves in those older than 65 years old and 70 years old for AVR and MVR, respectively [8]. But in the American College of Cardiology (ACC)/American Heart Association (AHA) guidelines, the age threshold for the selection of bioprosthetic valves is older than 65 for both AVR and MVR, while it is stated that either type of valve can be considered for AVR in patients between 50 and 65 years old [7]. Such inconsistency is also a consequence of limited clinical evidence for the optimal prosthesis for patients between 50 and 70 years.

Therefore, we performed a retrospective study based on real-world data of all patients aged 50–70 years who had undergone primary, isolated MVR or AVR in a national cardiac center in China between 2002 and 2007. The aim of this study was to compare long-term survival, stroke, and bleeding events in bioprosthetic versus mechanical valve replacement among patients aged between 50 and 70 years.

## 2. Materials and Methods

### 2.1 Overview

This was a single-center retrospective cohort study. The clinical information of patients who received isolated MVR or AVR in the Fuwai Hospital from 2002 to 2007 was collected. They were followed for at least 10 years, and the long-term survival rates of patients who received bioprosthetic or mechanical valve replacement were compared. And the results were reported according to the Strengthening the Reporting of Observational studies in Epidemiology (STROBE) Statement [9].

### 2.2 Inclusion and Exclusion Criteria

#### 2.2.1 Inclusion Criteria

Inclusion criteria were as follows:

1. The operation date was between January 1, 2002, and December 31, 2007;

2. 50 years old ≤ age ≤ 70 years old;

3. The patient received an isolated MVR or AVR.

#### 2.2.2 Exclusion Criteria

1. Patients died in the hospital or were discharged due to serious illness.

2. Patients underwent previous mitral or aortic replacement or repair.

3. Over 10% loss of any important items (demographic features, surgical information, and comorbidities) in the medical records.

4. Patients with concomitant coronary artery bypass surgery (CABG).

5. Patients undergoing emergency operation.

6. Patients with a history of drug abuse.

### 2.3 Surgical Methods and Type of prostheses

All patients underwent a median sternotomy under general anesthesia, cardiac valve replacement with cardiopulmonary bypass, and postoperative transesophageal echocardiography to assess the effects of valve replacement. Patients with bioprosthetic valves had routine warfarin anticoagulation for 6 months unless there were contraindications; patients with mechanical valves took warfarin for life. The bioprosthetic prostheses mainly included bovine pericardial valve (Carpentier-Edwards Perimount, Magna, Edwards Lifescience, Irvine, CA, USA), Hancock, Hancock II and Mosaic valves (Medtronic, Dublin, Ireland). The mechanical valve was mainly bileaftet valve (St. Jude, ATS, Medtronic, Sorin, Carbo). Tilting disk valve was also used in a small number of patients (10.11% (89/880) in mitral valve (MV) mechanical prostheses, 5.86% (19/324) in aortic valve (AV) mechanical prostheses).

### 2.4 Data Collection

Baseline information on demographics and co-morbidities, surgical procedures, and postoperative outcomes were obtained through the medical record system. Follow-up was conducted via telephone and letters by the surgical follow-up team of the Fuwai Hospital. For patients who could not be contacted by phone, the identity number registered on the front page of the medical record was used to perform a query on the resident death registration system. All the data used in this study were approved for scientific research and were not permitted for other purposes. Sensitive details of the patients were removed and patient information was kept strictly confidential.

### 2.5 Endpoints and Definition

The primary endpoint was all-cause mortality during the period from discharge to postoperative follow-up. Telephone and letter follow-ups were carried out until the death of the patient, and the patient was considered dead if the account was closed on the resident death registration system. The secondary endpoints included the incidence of stroke and major bleeding events. Incident stroke was defined as the first nonfatal or death due to ischemic, hemorrhagic, or iatrogenic stroke after valve surgery based on self-reporting or physician diagnosis. Major bleeding events included any intracerebral hemorrhage, gastrointestinal hemorrhage, hemarthrosis, retinal/choroidal hemorrhage, or receiving a blood transfusion. This was collected based on clinic visits and telephone calls. To minimize reporting discrepancies, these secondary endpoints events were reviewed and adjudicated by two senior clinicians (HS and ZZ) based on the documentation of each patient or presence of supporting laboratory or imaging results.

### 2.6 Statistical Analysis

Patient characteristics are presented as frequencies with percentages for categorical variables and as mean with standard deviation for continuous variables. To reduce selection bias, in both the AVR and the MVR cohorts, logistic regression was constructed respectively to generate the propensity score (PS). All baseline characteristics were included as covariates in the PS model in the AVR and MVR cohorts. Stabilized inverse probability-weighted (IPW) were calculated for each patient as the inverse of the PS for patients undergoing mechanical valves and the inverse of (1-PS) for patients undergoing bioprosthetic valves [10]. The balance between treatment groups was assessed with the use of standardized mean differences (SMD). A standardized difference of 10% or less was deemed to be the ideal balance [11]. An SMD of 20% or less was also considered acceptable.

Crude survival curves and IPW-adjusted curves for long-term survival were constructed in the MVR and AVR cohorts. The Kaplan–Meier method was used to calculate cumulative survival and curves were compared by the log-rank test. Since the effect of valve type is a time-varying variable, and the effect direction changed between 12 and 13 years of follow-up in the MVR cohort, but not the AVR cohort (**Supplementary Figs. 1,2**), a landmark analysis was performed to compare the long-term survival after 12.5 years in the MVR cohort [12]. The association of valve types with the primary and secondary outcomes were assessed in MVR and AVR cohorts and different age (50–59 years) and (60–70 years) groups using an unadjusted and adjusted hazard ratio (HR) by univariate, IPW weighted proportional model and multivariate cox proportional hazard models. Prespecified confounders included demographic features, body mass index (BMI), history of hypertension, hyperlipidemia, diabetes, chronic obstructive pulmonary disease, stroke, atrial fibrillation (AF), coronary heart disease, and New York Heart Association (NYHA) class were adjusted in the multivariate cox model. For additional subgroup analyses, we applied the multivariate cox model, controlling for other covariates other than the stratification variables, to evaluate the effect of mechanical valves on all-cause death in patients with different sex, BMI (divided by the median), AF, and NYHA class (I–II/III–IV) groups in both the MVR and AVR cohort and reported adjusted HR of mechanical valve compared with bioprosthetic valve in each subgroup. All tests were 2-tailed; an α level of 0.05 was considered statistically significant. All statistical analyses were performed using R software version 4.1.2 (R Foundation for Statistical Computing, Vienna, Austria).

## 3. Results

### 3.1 Study Population

**Fig. 1.**
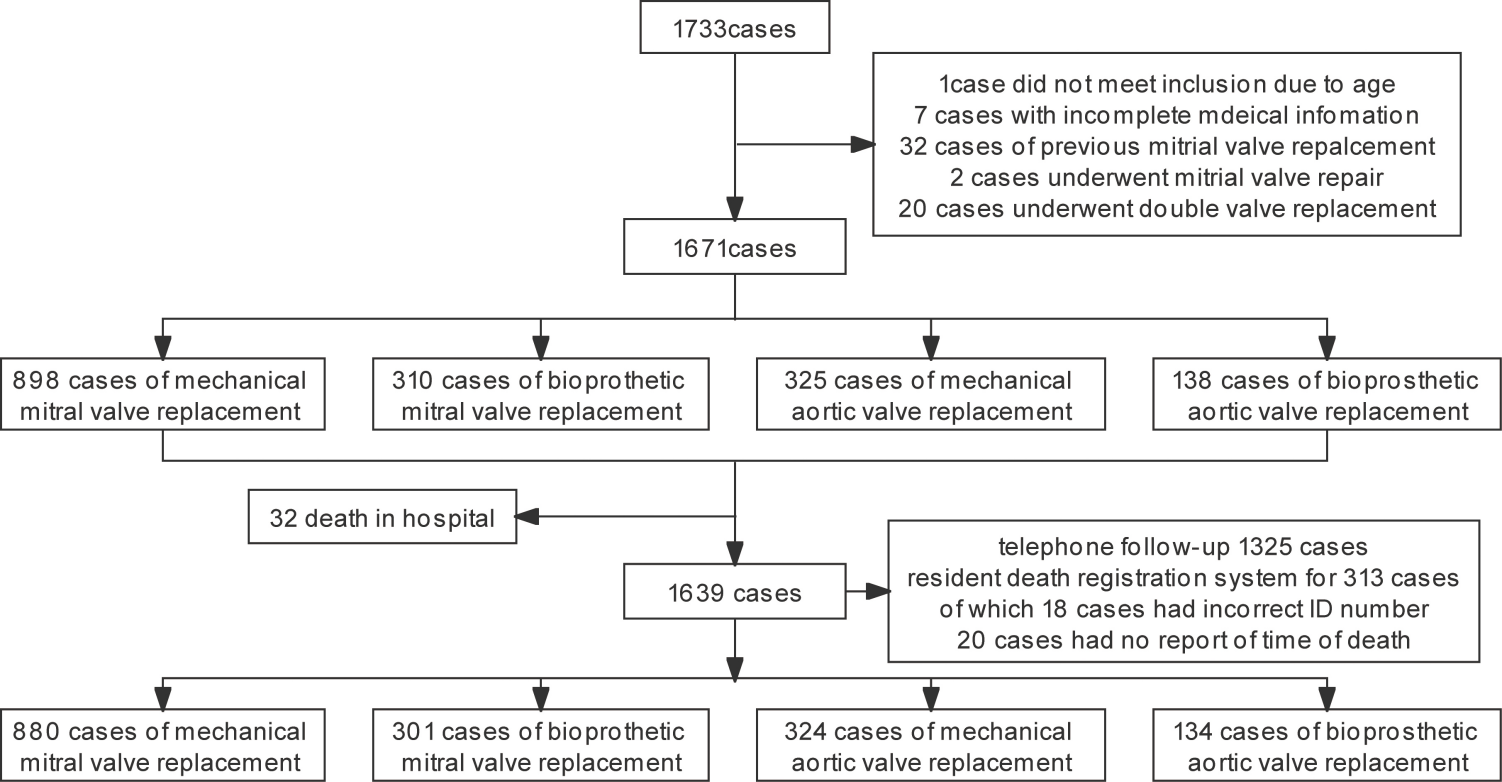
**Flow chart of patient enrollment**.

A total of 1733 cases met the inclusion criteria. The surgical records and homepage information of the 1733 cases were reviewed to exclude cases that did not meet the inclusion criteria (32 deaths occurred in hospital, including 17 patients who had a bioprosthetic MVR, 6 patients who had a mechanical MVR, 7 patients who had a mechanical AVR, and 2 patients who had a bioprosthetic AVR). A total of 1639 patients were enrolled in the study, including 1181 MVR patients (301 bioprosthetic valve replacements and 880 mechanical valve replacements) and 458 AVR patients (134 bioprosthetic valve replacements and 324 mechanical valve replacements) (Fig. 1).

In the unweighted analysis (**Supplementary Table 1**), patients who received a bioprosthetic valve were older than those who received a mechanical valve in both the MVR and AVR cohorts (mean [SD] age, 60.5 ± 5.3 years vs. 55.9 ± 4.4 years for MVR and 63.4 ± 5.0 years vs. 56.9 ± 4.9 years for AVR patients). In the MVR cohort, females accounted for approximately 70% of the population, while in the AVR cohort, over 60% of patients were male. Patients in the MVR cohorts were more likely to have AF compared with those with AVR. There was a small incidence of infective endocarditis in both the MVR (n = 9, 0.8%) and the AVR (n = 8, 1.7%) cohort, with a balanced distribution between the mechanical valve and bioprosthetic valve groups, both before and after IPW (**Supplementary Table 1** and Table 1). Other characteristics in both MVR and AVR cohorts were balanced after IPW with acceptable SMD lower than 0.2, except for hyperlipidemia in the AVR cohort (SMD = 0.2) (Table 1).

**Table 1.** **Baseline information after inverse-probability-weighted among MVR and AVR cohorts**.

		Mitral-valve replacement			Aortic-valve replacement		
		Bioprosthetic	Mechanical	SMD	Bioprosthetic	Mechanical	SMD
		(n = 1215.0)	(n = 1176.5)		(n = 455.4)	(n = 455.7)	
Age, yrs (mean (SD))		56.66 (5.37)	57.04 (4.98)	0.074	58.55 (6.03)	58.78 (5.73)	0.040
Female, n (%)		837.9 (69.0)	824.5 (70.1)	0.024	126.6 (27.8)	161.8 (35.5)	0.166
BMI (mean (SD))		23.50 (3.16)	23.53 (3.38)	0.011	24.63 (3.31)	24.45 (3.29)	0.055
Hypertension, n (%)		126.7 (10.4)	128.8 (10.9)	0.017	133.0 (29.2)	136.2 (29.9)	0.015
Hyperlipidemia, n (%)		40.7 (3.3)	38.6 (3.3)	0.004	0.0 (0.0)	9.0 (2.0)	0.200
Diabetes, n (%)		68.4 (5.6)	67.1 (5.7)	0.003	22.6 (5.0)	20.8 (4.6)	0.018
Stroke, n (%)		57.3 (4.7)	52.5 (4.5)	0.012	3.3 (0.7)	5.4 (1.2)	0.046
COPD, n (%)		71.9 (5.9)	90.6 (7.7)	0.071	38.6 (8.5)	29.8 (6.5)	0.073
PVD, n (%)		0.0 (0.0)	7.0 (0.6)	0.109	1.0 (0.2)	0.0 (0.0)	0.066
Infective endocarditis, n (%)		8.4 (0.7)	8.5 (0.7)	0.004	5.5 (1.2)	7.2 (1.6)	0.033
Atrial fibrillation, n (%)		912.8 (75.1)	884.1 (75.1)	<0.001	35.6 (7.8)	40.3 (8.8)	0.037
Coronary heart disease, n (%)		11.0 (0.9)	15.5 (1.3)	0.039	11.5 (2.5)	14.0 (3.1)	0.033
NYHA class, n (%)				0.168			0.123
	I	67.4 (5.5)	29.9 (2.5)		22.5 (4.9)	21.7 (4.8)	
	II	738.2 (60.8)	711.6 (60.5)		313.2 (68.8)	294.8 (64.7)	
	III	383.6 (31.6)	397.2 (33.8)		111.9 (24.6)	124.4 (27.3)	
	IV	25.8 (2.1)	37.8 (3.2)		7.7 (1.7)	14.7 (3.2)	
Liver disease, n (%)		2.6 (0.2)	3.2 (0.3)	0.011	0.0 (0.0)	1.0 (0.2)	0.066
Previous PCI, n (%)		1.0 (0.1)	0.0 (0.0)	0.041	3.2 (0.7)	2.2 (0.5)	0.027

Note: AVR, aortic valve replacement; MVR, mitral valve replacement; BMI, body mass index; COPD, chronic obstructive pulmonary disease; PVD, peripheral vascular disease; NYHA, New York Heart Association; PCI, percutaneous coronary intervention; SMD, standardized mean difference.

### 3.2 Bioprosthetic Valve versus Mechanical Valve 

The actual survival rate and relative risk after IPW or multivariate adjustment in primary and secondary outcomes among recipients of mechanical and biologic valve were compared in the MVR and AVR cohorts.

### 3.3 The MVR Cohort 

**Fig. 2.**
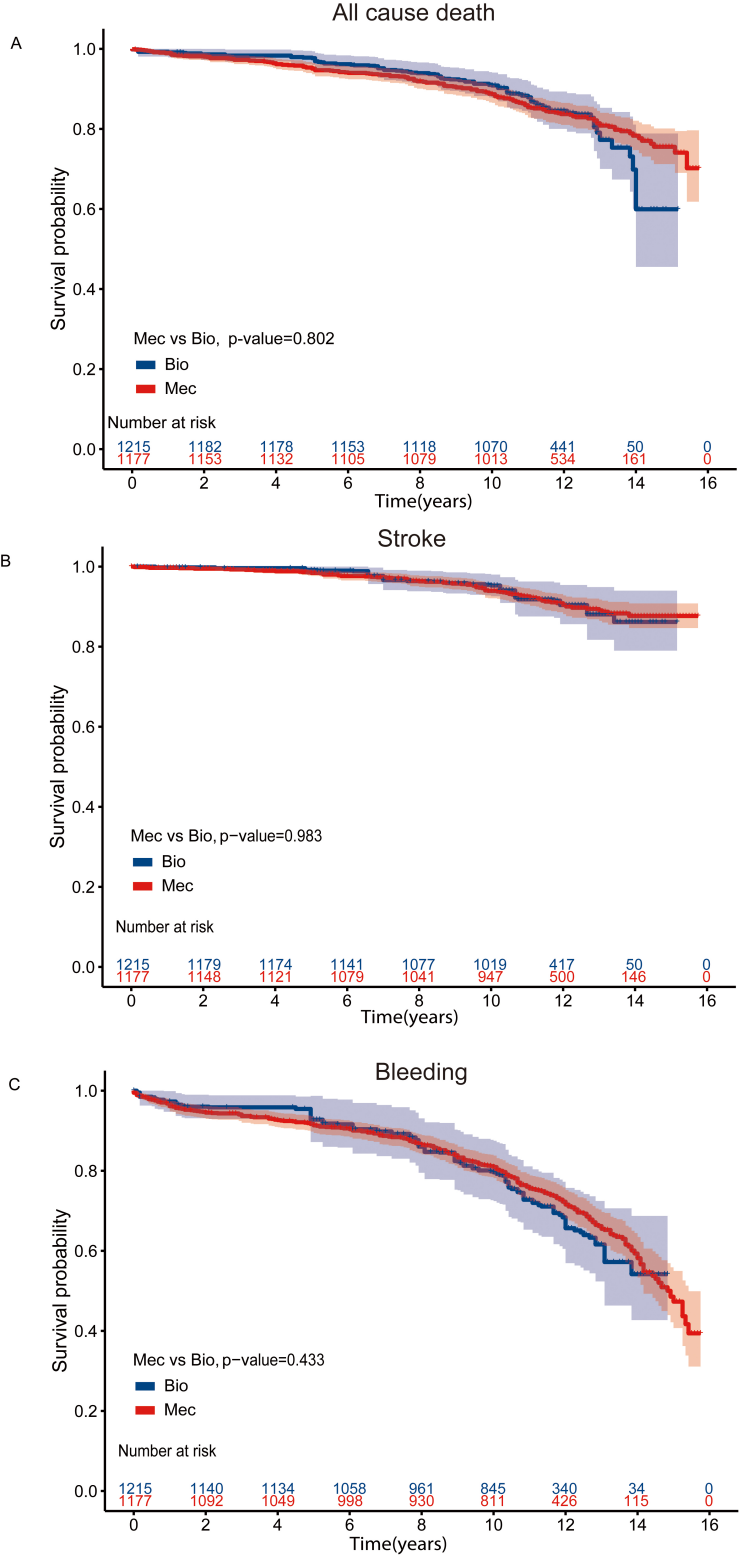
**Kaplan-Meier Curves for Clinical outcomes in the MVR cohort**. Kaplan-Meier curve of survival after IPW adjustment among patients aged 50–70 years who had undergone MVR (A) All cause death (B) Stroke events (C) Bleeding events. MVR, mitral valve replacement; IPW, inverse probability-weighted; Bio, bioprosthetic valves; Mec, mechanical values.

**Fig. 3.**
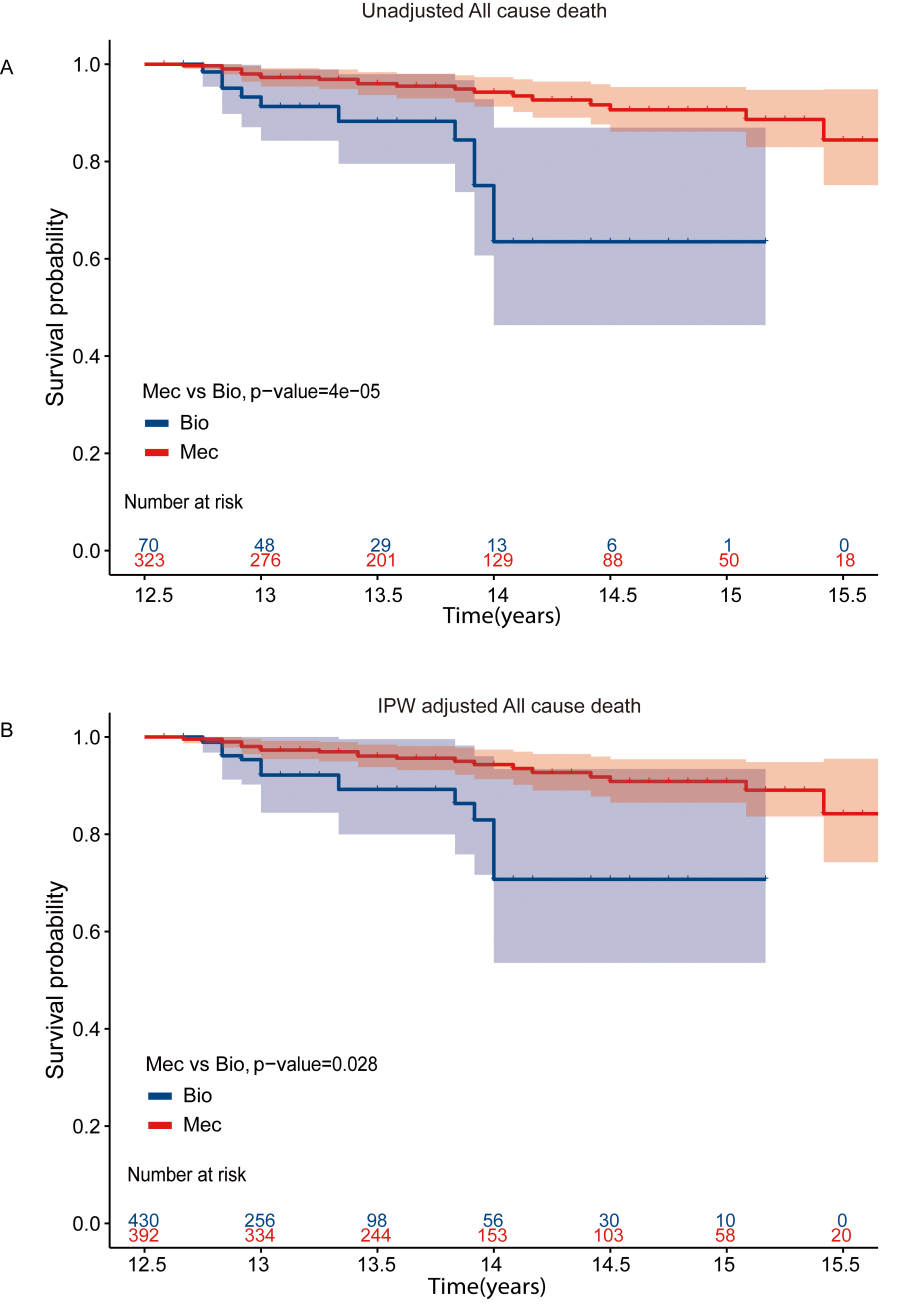
**Kaplan-Meier Curves for all cause mortality with landmark analysis in MVR cohort**. Kaplan-Meier curve of survival among patients aged 50–70 years with landmark analysis at 12.5 years in MVR cohort (A) Unadjusted survival curves (B) IPW adjusted survival curves. MVR, mitral valve replacement; IPW, inverse probability-weighted; Bio, bioprosthetic valves; Mec, mechanical values.

For MVR patients, the median follow-up period was 11.6 years and the 15-year survival rate was 48.3% and 75.7% for bioprosthetic valve and mechanical valve recipients respectively. Crude survival curves are shown in the **Supplementary Fig. 3**. After IPW adjustment, there was no statistically significant difference between long-term risk of mortality (Fig. 2A) [log-rank *p* = 0.802, HR (95% CI): 0.93 (0.66–1.31), *p* = 0.678], stroke (Fig. 2B) [log-rank *p* = 0.983, HR (95% CI): 0.99 (0.58–1.70); *p* = 0.967] and bleeding events (Fig. 2C) [log-rank *p* = 0.433, HR (95% CI): 0.88 (0.63–1.23), *p* = 0.467] among patients receiving mechanical valve replacement and bioprosthetic valve replacement (Table 2). However, after IPW adjustment (**Supplementary Table 2**), in the landmark analysis for comparing the survival rate after 12.5 years, we observed a higher survival rate in the mechanical MVR group (Fig. 3).

**Table 2.** **Between-Group Differences outcomes among Recipients of Mechanical and Biologic Valve in MVR cohort**.

	Mitral-valve replacement (50–70 yrs)			Mitral-valve replacement (50–59 yrs)			Mitral-valve replacement (60–70 yrs)		
	Bioprosthetic (n = 301)	Mechanical (n = 880)	*p*-value	Bioprosthetic (n = 129)	Mechanical (n = 703)	*p*-value	Bioprosthetic (n = 172)	Mechanical (n = 177)	*p*-value
Death	73 (48.3%)	152 (75.7%)	<0.001	22 (60.1%)	117 (76.8%)	0.5	51 (41.6%)	35 (71.7%)	0.020
(15-yrs survival rate)									
Crude model	ref	0.61 (0.46–0.81)	<0.001	ref	0.85 (0.54–1.35)	0.498	ref	0.61 (0.40–0.94)	0.023
HR (95% CI)									
IPW model	ref	0.93 (0.66–1.31)	0.678	ref	1.16 (0.70–1.93)	0.561	ref	0.66 (0.42–1.06)	0.085
HR (95% CI)									
Cox HR	ref	0.82 (0.59–1.12)	0.207	ref	0.98 (0.61–1.58)	0.937	ref	0.71 (0.44–1.14)	0.16
HR (95% CI)									
Stroke (percentage)	27 (9.0%)	74 (8.4%)	0.400	10 (7.8%)	60 (8.5%)	0.900	17 (9.9%)	14 (7.9%)	0.300
Crude model	ref	0.84 (0.54–1.30)	0.428	ref	1.03 (0.53–2.02)	0.928	ref	0.71 (0.35–1.44)	0.339
HR (95% CI)									
IPW model	ref	0.99 (0.58–1.70)	0.967	ref	0.92 (0.45–1.90)	0.824	ref	0.90 (0.43–1.90)	0.788
HR (95% CI)									
Cox HR	ref	1.01 (0.62–1.64)	0.982	ref	1.11 (0.56–2.21)	0.762	ref	0.85 (0.40–1.83)	0.682
HR (95% CI)									
Bleeding (percentage)	73 (24.3%)	266 (30.2%)	0.500	38 (29.5%)	215 (30.6%)	0.600	35 (20.3%)	51 (28.8%)	0.200
Crude model	ref	1.09 (0.84–1.41)	0.530	ref	0.92 (0.65–1.30)	0.629	ref	1.316 (0.85–2.03)	0.213
HR (95% CI)									
IPW model	ref	0.88 (0.63–1.23)	0.467	ref	0.87 (0.59–1.27)	0.471	ref	1.18 (0.75–1.86)	0.483
HR (95% CI)									
Cox HR	ref	1.06 (0.79–1.41)	0.710	ref	0.93 (0.65–1.33)	0.683	ref	1.18 (0.74–1.89)	0.488
HR (95% CI)									

MVR, mitral valve replacement; HR, hazard ratio; CI, confidence interval; Crude model HR, analyzed in the univariate model, inverse probability-weighted (IPW) model HR, analyzed in IPW model; Cox HR: adjusted for demographic features, body mass index (BMI), history of hypertension, hyperlipidemia, diabetes, chronic obstructive pulmonary disease (COPD), stroke, atrial fibrillation (AF), coronary heart disease (CHD) and New York Heart Association (NYHA) class. ref refers to the reference in the model.

### 3.4 The AVR Cohort

**Fig. 4.**
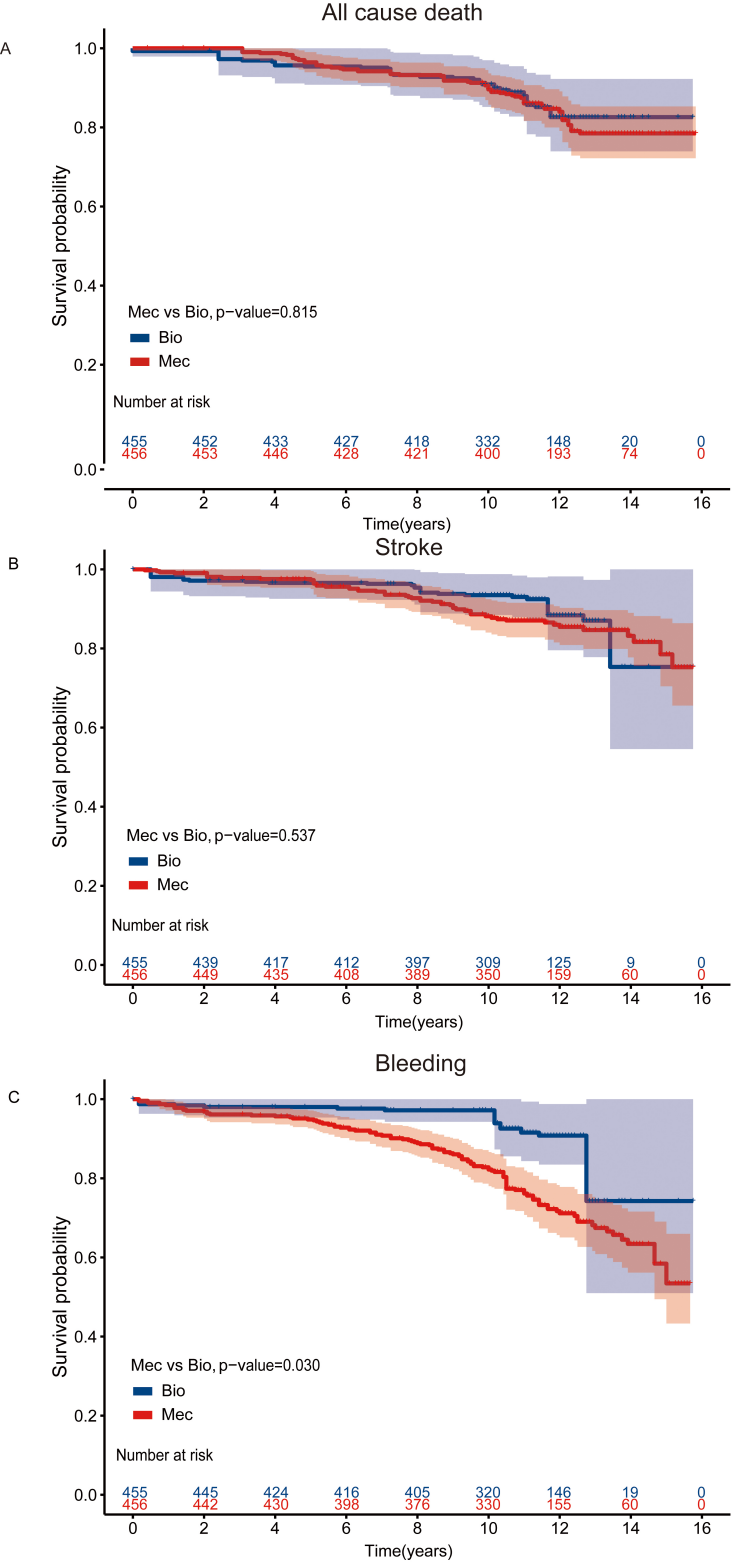
**Kaplan-Meier Curves for Clinical outcomes in the AVR cohort**. Kaplan-Meier curve of survival after IPW adjustment among patients aged 50–70 years who had undergone AVR (A) All cause death (B) Stroke events (C) Bleeding events. AVR, aortic valve replacement; IPW, inverse probability-weighted; Bio, bioprosthetic valves; Mec, mechanical values.

For AVR patients, the median follow-up time was 11.4 years, with a 15-year survival rate of 80.4% and 81.0% in the bioprosthetic and mechanical valve groups, respectively. Crude survival curves are shown in **Supplementary Fig. 4**. The long-term risk of mortality (Fig. 4A) and stroke (Fig. 4B) in the two types of valve among AVR patients was not significantly different [Mortality: log-rank *p* = 0.815, HR (95% CI): 1.12 (0.6–2.09), *p* = 0.725; Stroke: log-rank *p* = 0.537, HR (95% CI): 1.39 (0.64–3.02), *p* = 0.405]. Patients receiving mechanical valves had a higher risk of bleeding (Fig. 4C) compared with those with bioprosthetic valves [log-rank *p* = 0.03, HR (95% CI): 2.52 (1.06–5.93), *p* = 0.036] (Table 3).

**Table 3.** **Between-Group Differences outcomes among Recipients of Mechanical and Biologic Valve in AVR cohort**.

	Aortic–valve replacement (50–70 yrs)			Aortic–valve replacement (50–59 yrs)			Aortic–valve replacement (60–70 yrs)		
	Bioprosthetic (n = 134)	Mechanical (n = 324)	*p*-value	Bioprosthetic (n = 33)	Mechanical (n = 237)	*p*-value	Bioprosthetic (n = 101)	Mechanical (n = 87)	*p*-value
Death	23 (80.4%)	49 (81.0%)	0.500	6 (77.0%)	34 (83.0%)	0.500	17 (81.9%)	15 (73.2%)	1.000
(15–yrs survival rate)									
Crude model	ref	0.83 (0.51–1.37)	0.469	ref	0.73 (0.31–1.75)	0.482	ref	1.00 (0.50–2.00)	1.000
HR (95% CI)									
IPW model	ref	1.12 (0.60–2.09)	0.725	ref	1.00 (0.35–2.92)	0.996	ref	1.11 (0.54–2.27)	0.777
HR (95% CI)									
Cox HR	ref	0.84 (0.45–1.56)	0.588	ref	0.55 (0.21–1.44)	0.222	ref	1.12 (0.56–2.25)	0.742
HR (95% CI)									
Stroke (percentage)	16 (11.9%)	45 (13.9%)	0.999	3 (9.1%)	34 (14.3%)	0.6	13 (12.9%)	11 (12.6%)	0.800
Crude model	ref	1.02 (0.57–1.82)	0.950	ref	1.35 (0.41–4.41)	0.623	ref	0.92 (0.41–2.07)	0.842
HR (95% CI)									
IPW model	ref	1.39 (0.64–3.02)	0.405	ref	2.02 (0.54–7.56)	0.296	ref	1.31 (0.53–3.22)	0.554
HR (95% CI)									
Cox HR	ref	1.09 (0.54–2.18)	0.808	ref	1.098 (0.34–3.60)	0.878	ref	1.69 (0.75–3.80)	0.203
HR (95% CI)									
Bleeding (percentage)	10 (7.5%)	97 (29.9%)	<0.001	4 (12.1%)	76 (32.1%)	0.06	6 (5.9%)	21 (24.1%)	<0.001
Crude model	ref	3.97 (2.07–7.62)	<0.001	ref	2.54 (0.93–6.95)	0.07	ref	4.31 (1.74–10.71)	0.002
HR (95% CI)									
IPW model	ref	2.51 (1.06–5.93)	0.036	ref	2.11 (0.76–6.23)	0.176	ref	3.57 (1.36–9.36)	0.010
HR (95% CI)									
Cox HR	ref	3.07 (1.51–6.24)	0.002	ref	2.50 (0.88–7.09)	0.084	ref	3.48 (1.29–9.41)	0.014
HR (95% CI)									

AVR, aortic valve replacement; HR, hazard ratio; CI, confidence interval; Crude model HR, analyzed in the univariate model, inverse probability-weighted (IPW) model HR, analyzed in IPW model; Cox HR: adjusted for demographic features, body mass index (BMI), history of hypertension, hyperlipidemia, diabetes, chronic obstructive pulmonary disease (COPD), stroke, atrial fibrillation (AF), coronary heart disease (CHD) and New York Heart Association (NYHA) class. ref refers to the reference in the model.

### 3.5 Subgroup Analysis

**Fig. 5.**
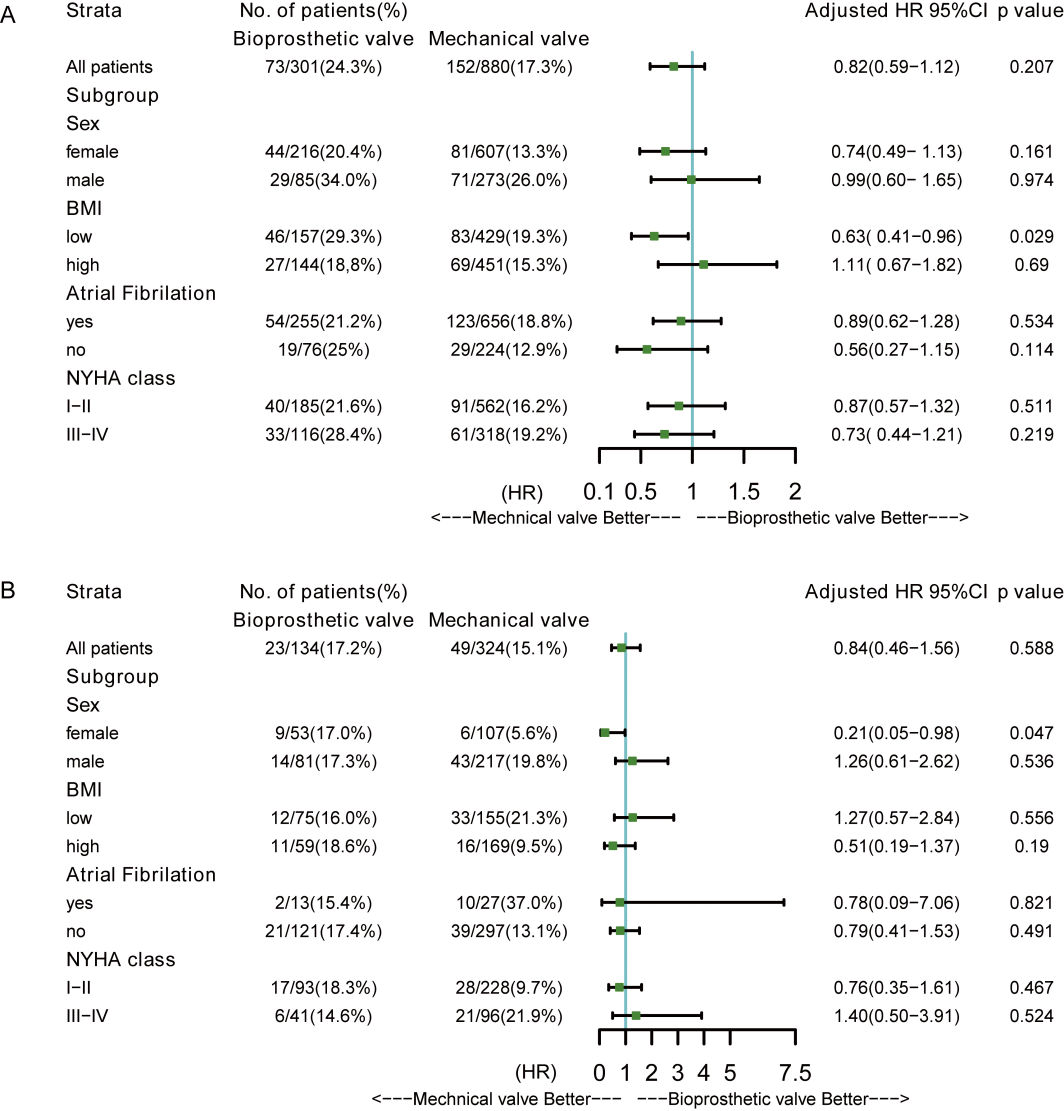
**Forest plots for subgroup analysis**. (A) Multivariate adjusted HR of mechanical valves versus bioprosthetic valves in MVR cohort. (B) Multivariate adjusted HR of mechanical valves versus bioprosthetic valves in AVR cohort. AVR, aortic valve replacement; MVR, mitral valve replacement; HR, hazard ratio; BMI, body mass index; NYHA, New York Heart Association.

In the subgroup analysis, patients receiving a valve replacement were stratified into “50 ≤ age ≤ 59 years old” and “60 ≤ age ≤ 70 years old” subgroups. IPW were performed in each age group to balance the baseline characteristics (**Supplementary Tables 3,4**). For patients undergoing MVR, there was no statistically significant difference between patients receiving mechanical valves and bioprosthetic valves in the long-term risk of mortality, stroke, and bleeding events in both age subgroups [50–59, HR (95% CI) mortality: 1.16 (0.70–1.93), *p* = 0.561; stroke 0.92 (0.45–1.90), *p* = 0.824; for bleeding 0.87 (0.59–1.27), *p* = 0.471; 60–70 HR (95% CI) mortality: 0.66 (0.42–1.06), *p* = 0.085; stroke 0.90 (0.43–1.90), *p* = 0.788; bleeding 1.18 (0.75–1.86), *p* = 0.483] (Table 2). For patients undergoing AVR, there was no statistically significant difference between the two types of prosthesis in mortality and stroke risk for both age groups, but the bleeding risk in patients receiving a mechanical AVR was significantly higher in patients between 60 and 70 years old [HR (95% CI): 3.57 (1.36–9.36), *p* = 0.010] (Table 3). The forest plots illustrate the multivariate-adjusted effect of valve types on the primary outcome in the other AVR and MVR subgroups. Among patients with a low BMI (below the median) in the MVR cohort, mechanical valves were associated with a significantly lower risk of mortality compared with bioprosthetic valves [HR (95% CI): 0.63 (0.41–0.96)] (Fig. 5A). In the AVR cohort, mechanical valves were associated with a significantly lower risk of mortality in the female group [HR (95% CI): 0.21(0.05–0.98)] (Fig. 5B).

## 4. Discussion

Based on a large series of isolated MVR and AVR cohorts, the present study did not find a significant difference using IPW in overall survival rates and stroke rates between mechanical valves and bioprosthetic valves among patients between 50 and 70 years old in both MVR and AVR cohorts. However, landmark analysis revealed a significantly lower mortality in patients receiving a mechanical MVR after 12.5 years. In the AVR cohort, bioprosthetic valves were associated with a significantly lower risk of bleeding events.

In the IPW adjustment for the MVR cohorts, all the baseline characteristics were well-balanced in the MVR except for NYHA class, which had the SMD of 16.8%. We did not observe a significant difference in mortality between the mechanical and bioprosthetic groups, although the HR point estimates trended towards a benefit for mechanical valves [HR (95% CI): 0.93 (0.66–1.31)]. For the sensitivity analysis, we also performed a multivariate adjustment by the Cox model, and found that the HR of mechanical valves was in the same direction but without statistical significance [HR (95% CI): 0.82 (0.59–1.12)]. These results are in line with the previous results reported by Chikwe *et al*. [13]. In their retrospective cohort analysis of 3433 patients aged 50–69 years who underwent primary MVR in a single center, they found no survival difference at 15 years between the use of mechanical and bioprosthetic mitral valves [HR (95% CI): 0.95 (0.79–1.15)]. Another study of 8015 MVR patients aged 50–69 and a longer-follow-up time, found a relatively higher 15-year mortality in recipients of biologic prostheses [HR (95% CI): 1.16 (1.04–1.30)] [14]. In this study, we only included patients with isolated MVR and excluded those patients undergoing other valve surgeries and CABG. The point estimate of HR showed a similar direction, indicating bioprosthetic valves might be related to unsatisfactory long-term outcomes. This might be due to the shorter durability of bioprosthetic valves, which only last between 10–15 years [6, 15]. We also found that in the MVR cohorts, the valve type was a time-varying variable and the effect direction changed between 12 and 13 years of follow-up (**Supplementary Fig. 1**). Therefore, we employed a landmark analysis with the landmark time of 12.5 years. In the crude analysis and IPW adjustment, after a follow-up of 12.5 years, patients with mechanical valves had a higher survival rate, indicating that at least for those reaching the landmark year, mechanical valves for MVR might be associated with benefits over the bioprosthetic valves. This finding supports evidence for the new ESC guideline recommendations which states that mechanical protheses should be considered for those with a reasonable life expectancy and would be at risk for undergoing future valve surgery [8].

Among the current publications comparing mechanical and bioprosthetic valves, the present study is unique for using IPW to compare the outcomes of the two types of valves in the isolated MVR and AVR cohorts based on a relatively large sample size with long-term follow-up in the Chinese population. Although accumulating studies focused on the issue of valve selection in patients between 50 and 70 years [4, 16, 17], the results were mainly derived from western populations with limited evidence from Asian populations. The clinical demographics of Asian populations are quite distinct from the western population as seen in our study. There are more female patients, and a higher proportion of AF in both the mechanical and bioprosthetic MVR cohort, suggesting that the recipients of bioprosthetic valves had more risk factors. Prior to 2010, guidelines (for example ACC/AHA 2006) recommended a similar targeted range of international normalized ratio of 2.5–3.5 for warfarin among both those receiving mechanical MVR and bioprosthetic MVR with risk factors [18]. Therefore, the distinct population features in our study might be a possible explanation for our observed comparable rates of bleeding events between the two types of valves, in contrast to previously reported significantly lower bleeding rates in the bioprosthetic MVR group.

In the AVR group, no difference was observed in survival rate or stroke rate between recipients of mechanical and bioprosthetic valves, but the mechanical valves were associated with a higher likelihood of bleeding. Similar results were found by Chiang and colleagues in 4253 patients aged 50–69 years who underwent primary isolated AVR [19]. The age and sex distribution in that study was similar to ours. After PS matching, 1001 patients were paired, and the HR for death, stroke and bleeding in mechanical versus biologic prostheses were 0.97 (95% CI, 0.83–1.14), 1.04 (95% CI, 0.75–1.43) and 1.75 (95% CI, 1.27–2.43) respectively. Glaser reported a lower risk of major bleeding events [HR (95% CI): 0.49 (0.34–0.70)] in the bioprostheses group and a non-significant difference in stroke risk [HR (95% CI): 1.04 (0.72–1.50)] [20]. Despite the absence of a significant survival benefit, the higher risk of bleeding events should be considered for valve selection in an Asian population with a relatively low burden of comorbidities undergoing isolated AVR.

It is also necessary to note that the low low re-operation rate in the bioprosthetic group might also be associated with increased long-term mortality. Due to the concerns about surgical risk and economic factors, patients are not that active in the second operation. Many people choose supportive treatment. In our study, only 4 patients (2.98%) in the AVR bioprosthetic valve group and 9 patients (2.99%) in the MVR bioprosthetic valve group receive second operation for new bioprostheic valve due to the valve failure, none of whom died during the follow-up. As an alternative to surgical valve replacement, transcatheter aortic valve replacement (TAVR) is also an emerging strategy for bioprosthetic valve replacement in the intermediate-to-low-risk population. Recent studies have also indicated that TAVR is a safe procedure, with low rates of in hospital death and severe complications in mid-term follow-up for patients under 70 years old [21]. Therefore, in the population between 50 and 70 years old, a TAVR might be a reasonable choice considering the lower rate of major bleeding events and surgical risk. Furthermore, valve-in-valve technology was introduced as an alternative to surgical valve replacement opportunities to patients with valve failure after bioprosthetic valve replacement. Retrospective studies have also demonstrated early benefits of valve in valve technique in patients presenting with failed aortic and mitral bioprostheses [22, 23]. But more data, longer follow-up times, and multicenter studies are needed in the future to evaluate its efficiency, efficacy and benefits compared with the surgical valve replacement.

We explored the primary and secondary outcomes in different age subgroups and did not observe a significant difference between the mechanical and prosthetic groups in both the MVR and AVR cohorts except for a higher risk of bleeding in those older patients (60–70 years old) receiving mechanical aortic valves. In subgroups, after multivariate adjustment, we found a relatively lower risk of mortality in mechanical valve recipients compared with those receiving bioprosthetic valves in those undergoing MVR with a lower BMI (less than 23.4 kg/$m^{2}$) and female patients undergoing AVR, which suggests a potential effect of BMI and sex on the treatment effect of different types of valves. Since the events are limited in the AVR group stratified by different sex, we further explored BMI in relation to valve types in the MVR cohort into four groups according to the median of BMI and valve types. In a survival analysis, we found that those patients with low BMI and bioprosthetic mitral valves had a relatively lower survival rate (log-rank *p* < 0.001) (**Supplementary Fig. 5**). Given the previous discussion on the impact of BMI or sex on outcomes after valve surgery [24, 25], these results, although from a post-hoc exploratory analysis for hypothesis generating, might also be an indicator for future studies to consider the impact of these factors on valve selection in relation to the outcomes of MVR or AVR.

Compared to other studies analyzing patients with valve replacement, our study has the longest follow-up and the largest sample size in China. Given that patients in a national center come from a variety of areas and are associated with diverse demographic characteristics, our findings may, to a certain extent, reflect and represent the overall state of long-term survival rate of patients following valve replacement in China. Both IPW and multivariate cox models were used to adjust the cofounders to strengthen the results.

In any retrospective study, there may be residual confounding owing to unmeasured variables although the inverse probability weighting method addressed the issue of selection bias. The main outcomes evaluated in this study were long-term mortality as well as stroke and bleeding events following surgery, while the data related to postoperative valve failure and reoperation, or quality of life were limited. Furthermore, since the sample size in the AVR cohort was limited, there might be a risk of overfitting in the multivariate model in the subgroup analysis. Though the primary outcomes were obtained through telephone contact or query on the resident death registration system, we could not rule out the possibility that patients who were lost during the long-term follow-up had experienced subsequent events that were not captured in our study.

## 5. Conclusions

In summary, in this study we did not observe significant differences in the long-term survival rates and stroke rates of Chinese patients aged 50–70 with bioprosthetic or mechanical valves for MVR or AVR. However, those mechanical mitral valve recipients who were followed for over 12.5 years showed a lower mortality while the recipients of bioprosthetic aortic valves displayed a lower risk for long-term bleeding events. The life expectancy and risk of undergoing future valve surgery should be considered when selecting the biroprosthetic valves for MVR while the need for anticoagulation medication and the risk of bleeding should not be ignored when selecting mechanical aortic valves. Additional long-term follow-up is needed to more adequately assess the lifetime risks of different types of valve prostheses.

## Data Availability

All data generated or analyzed during this study are included in this published article.

## Supplementary Material

Supplementary material associated with this article can be found, in the online version, at https://doi.org/10.31083/j.rcm2409253.
