# Supplementary material for: Long-Term Outcomes of Bioprosthetic and Mechanical Valve Replacement for Patients Aged between 50 and 70 Years
Source: Rev Cardiovasc Med. 2023 Sep 18;24(9):253. doi: 10.31083/j.rcm2409253 (PMC11270086; doi:10.31083/j.rcm2409253)
Supplement: Supplementary file 1 [file 2153-8174-24-9-253-s1.zip › 2153-8174-24-9-253-s1.docx]

#### Supplementary materials

**Long-term outcomes of bioprosthetic and mechanical valve replacement for patients aged between 50 and 70 years**

**Authors**: Wei Zhao, MD, ^1,2†^ ,Zhongli Chen, MD^5†^, Sipeng Chen，MPH ^1,3†^ ,Junzhe Du, MD, PhD ^1,2,4^ ,Heng Zhang, MD, PhD ^1,2^ ,Yan Zhao , MD, ^1,^Li He, MD, ^1,^ Wei Feng, MD, PhD ^1,2^ ,Hansong Sun MD, PhD ^1,2^ ,Zhe Zheng, MD, PhD^1,2^

**Corresponding Author:** Dr. Zhe Zheng

Fuwai Hospital, 167 Beilishi Road, Beijing 100037, People’s Republic of China; Tel: +86 10 8839 6051; Fax: +86 10 8839 6051; E-mail: [zhengzhe@fuwai.com](mailto:zhengzhe@fuwai.com)

^†^These authors contributed equally.

**SUPPLEMENTARY MATERIAL CONTENT**

Table S1-S4

Figure S1: Relationship between effect of valve types and time in MVR cohort.

Figure S2: Relationship between effect of valve types and time in AVR cohort.

Figure S3: Unadjusted Kaplan-Meier curve of survival among patients aged 50 – 70 years who had undergone MVR (A) all cause death (B) Stroke events (C) Bleeding events

Figure S4: Unadjusted Kaplan-Meier curve of survival among patients aged 50 – 70 years who had undergone AVR (A) all cause death (B) Stroke events (C) Bleeding events

Figure S5: Unadjusted Kaplan-Meier curve of survival among patients aged 50 – 70 years stratified by BMI and valve types in MVR cohort.

**Table S1 Unadjusted baseline information of MVR and AVR patients**

|  | | |  | **Mitral-Valve Replacement** | | | **Aortic-Valve Replacement** | | | |
| --- | --- | --- | --- | --- | --- | --- | --- | --- | --- | --- |
|  | | **All**  **(n=1181)** | | **Bioprosthetic (n=301)** | **Mechanical**  **(n=880)** | SMD | **All**  **(n=458)** | **Bioprosthetic (n=134)** | **Mechanical**  **(n=324)** | SMD |
| **Age,yrs (mean (SD))** | 57.1 (5.1) | | | 60.48 (5.3) | 55.93 (4.4) | 0.929 | 58.8 (5.8) | 63.42 (5.0) | 56.89 (4.9) | 1.313 |
| **Female, n (%)** | 823 (69.7) | | | 216 (71.8) | 607 (69.0) | 0.061 | 160 (34.9) | 53 (39.6) | 107 (33.0) | 0.136 |
| **BMI (mean (SD))** | 23.5 (3.3) | | | 23.38 (3.2) | 23.55 (3.4) | 0.051 | 24.4 (3.3) | 23.79 (3.3) | 24.67 (3.3) | 0.270 |
| **Hypertension, n (%)** | 129 (10.9) | | | 39 (13.0) | 90 (10.2) | 0.085 | 142 (31.0) | 51 (38.1) | 91 (28.1) | 0.213 |
| **Hyperlipidemia, n (%)** | 38 (3.2) | | | 10 (3.3) | 28 (3.2) | 0.008 | 9 (2.0) | 0 (0.0) | 9 (2.8) | 0.239 |
| **Diabetes, n (%)** | 66 (5.6) | | | 19 (6.3) | 47 (5.3) | 0.041 | 26 (5.7) | 12 (9.0) | 14 (4.3) | 0.187 |
| **stroke, n (%)** | 54 (4.6) | | | 21 (7.0) | 33 (3.8) | 0.144 | 6 (1.3) | 2 (1.5) | 4 (1.2) | 0.022 |
| **COPD, n (%)** | 93 (7.9) | | | 26 (8.6) | 67 (7.6) | 0.037 | 26 (5.7) | 11 (8.2) | 15 (4.6) | 0.146 |
| **PVD, n (%)** | 7 (0.6) | | | 0 (0.0) | 7 (0.8) | 0.127 | 1 (0.2) | 1 (0.7) | 0 (0.0) | 0.123 |
| **Infective endocarditis, n (%)** | 9 (0.8) | | | 2 (0.7) | 7 (0.8) | 0.015 | 8(1.7) | 2 (1.5) | 6 (1.9) | 0.028 |
| **Atrial fibrillation, n (%)** | 881 (74.6) | | | 225 (74.8) | 656 (74.5) | 0.005 | 17 (3.7) | 13 (9.7) | 27 (8.3) | 0.048 |
| **Coronary heart disease, n (%)** | 18 (1.5) | | | 6 (2.0) | 12 (1.4) | 0.049 | 40 (8.7) | 7 (5.2) | 10 (3.1) | 0.107 |
| **NYHA class, n (%)** |  | | |  |  | 0.113 |  |  |  | 0.098 |
| **I** | 30 (2.5) | | | 5 (1.7) | 25 (2.8) |  | 20 (4.4) | 6 (4.5) | 14 (4.3) |  |
| **II** | 717 (60.7) | | | 180 (59.8) | 537 (61.0) |  | 301 (65.7) | 87 (64.9) | 214 (66.0) |  |
| **III** | 394 (33.4) | | | 108 (35.9) | 286 (32.5) |  | 122 (26.6) | 38 (28.4) | 84 (25.9) |  |
| **IV** | 40 (3.4) | | | 8 (2.7) | 32 (3.6) |  | 15 (3.3) | 3 (2.2) | 12 (3.7) |  |
| **Liver disease, n (%)** | 3 (0.3) | | | 1 (0.3) | 2 (0.2) | 0.020 | 1 (0.2) | 0 (0.0) | 1 (0.3) | 0.079 |
| **Previous PCI, n (%)** | 1 (0.1) | | | 1 (0.3) | 0 (0.0) | 0.082 | 3 (0.7) | 2 (1.5) | 1 (0.3) | 0.126 |

*Note: BMI, body mass index; COPD, chronic obstructive pulmonary disease; PVD, peripheral vascular disease; NYHA, New York Heart Association; PCI, Percutaneous Coronary Intervention; SMD, standardized mean difference*

**Table S2 Baseline information after inverse-probability-weighted among different age groups in the MVR cohort.**

|  | **Mitral-Valve Replacement IPW**  **(50-59 yrs)** | | | **Mitral-Valve Replacement IPW**  **(60-70 yrs)** | | |
| --- | --- | --- | --- | --- | --- | --- |
|  | **Bioprosthetic  (n=837.9)** | **Mechanical (n=831.6)** | **SMD** | **Bioprosthetic  (n=344.3)** | **Mechanical (n=351.2)** | **SMD** |
| **Age,yrs (mean (SD))** | 54.10 (3.1) | 54.33 (2.8) | 0.079 | 63.66 (2.8) | 63.71 (2.7) | 0.018 |
| **Female, n (%)** | 587.4 (70.1) | 585.1 (70.4) | 0.006 | 239.4 (69.5) | 241.3 (68.7) | 0.018 |
| **BMI (mean (SD))** | 23.48 (3.3) | 23.53 (3.3) | 0.014 | 23.52 (3.1) | 23.43 (3.7) | 0.027 |
| **Hypertension, n (%)** | 82.3 (9.8) | 70.2 (8.4) | 0.048 | 56.4 (16.4) | 61.2 (17.4) | 0.028 |
| **Hyperlipidemia, n (%)** | 30.2 (3.6) | 31.1 (3.7) | 0.007 | 7.3 (2.1) | 7.8 (2.2) | 0.007 |
| **Diabetes, n (%)** | 27.4 (3.3) | 34.5 (4.1) | 0.046 | 29.0 (8.4) | 30.7 (8.7) | 0.012 |
| **stroke, n (%)** | 36.1 (4.3) | 38.1 (4.6) | 0.014 | 15.8 (4.6) | 14.7 (4.2) | 0.020 |
| **COPD, n (%)** | 51.0 (6.1) | 62.5 (7.5) | 0.057 | 28.5 (8.3) | 34.5 (9.8) | 0.053 |
| **PVD, n (%)** | 0.0 (0.0) | 6.0 (0.7) | 0.121 | 0.0 (0.0) | 1.0 (0.3) | 0.076 |
| **Infective endocarditis, n (%)** | 7.3 (0.9) | 8.0 (1.0) | 0.010 | 1.0 (0.3) | 0.0 (0.0) | 0.076 |
| **Atrial fibrillation, n (%)** | 590.8 (70.5) | 612.4 (73.6) | 0.070 | 265.1 (77.0) | 269.0 (76.6) | 0.009 |
| **Coronary heart disease, n (%)** | 0.0 (0.0) | 6.0 (0.7) | 0.121 | 9.6 (2.8) | 9.9 (2.8) | 0.002 |
| **NYHA class, n (%)** |  |  | 0.130 |  |  | 0.073 |
| **I** | 37.6 (4.5) | 22.1 (2.7) |  | 4.0 (1.2) | 7.2 (2.1) |  |
| **II** | 526.3 (62.8) | 506.5 (60.9) |  | 204.4 (59.4) | 203.4 (57.9) |  |
| **III** | 255.1 (30.4) | 274.1 (33.0) |  | 127.5 (37.0) | 132.0 (37.6) |  |
| **IV** | 19.0 (2.3) | 28.8 (3.5) |  | 8.4 (2.4) | 8.5 (2.4) |  |
| **Liver disease, n (%)** | 0.0 (0.0) | 1.0 (0.1) | 0.049 | 2.1 (0.6) | 2.1 (0.6) | 0.002 |
| **Previous PCI, n (%)** | 0.0 (0.0) | 0.0 (0.0) | <0.001 | 1.0 (0.3) | 0.0 (0.0) | 0.076 |

*Note: BMI, body mass index; COPD, chronic obstructive pulmonary disease; PVD, peripheral vascular disease; NYHA, New York Heart Association; PCI, Percutaneous Coronary Intervention; SMD, standardized mean difference*

**Table S3 Baseline comparison in after inverse-probability-weighted in 12.5-year landmark MVR cohort.**

|  | **Mitral-Valve Replacement crude** | | | **Mitral-Valve Replacement IPW** | | |
| --- | --- | --- | --- | --- | --- | --- |
|  | **Bioprosthetic  (n=70)** | **Mechanical (n=323)** | **SMD** | **Bioprosthetic  (n=429.9)** | **Mechanical (n=391.6)** | **SMD** |
| **Age,yrs (mean (SD))** | 59.60 (5.0) | 55.25 (3.9) | 0.964 | 55.15 (4.9) | 56.02 (4.5) | 0.186 |
| **Female, n (%)** | 48 (68.6) | 222 (68.7) | 0.003 | 290.4 (67.5) | 270.5 (69.1) | 0.033 |
| **BMI (mean (SD))** | 22.95 (2.7) | 23.68 (3.1) | 0.253 | 23.64 (2.7) | 23.54 (3.1) | 0.032 |
| **Hypertension, n (%)** | 5 (7.1) | 20 (6.2) | 0.038 | 13.5 (3.1) | 24.2 (6.2) | 0.145 |
| **Hyperlipidemia, n (%)** | 2 (2.9) | 9 (2.8) | 0.004 | 9.4 (2.2) | 10.9 (2.8) | 0.038 |
| **Diabetes, n (%)** | 2 (2.9) | 17 (5.3) | 0.122 | 19.6 (4.6) | 19.2 (4.9) | 0.016 |
| **stroke, n (%)** | 4 (5.7) | 7 (2.2) | 0.183 | 8.5 (2.0) | 10.6 (2.7) | 0.048 |
| **COPD, n (%)** | 7 (10.0) | 24 (7.4) | 0.091 | 26.7 (6.2) | 30.2 (7.7) | 0.059 |
| **PVD, n (%)** | 0 (0.0) | 3 (0.9) | 0.137 | 0.0 (0.0) | 3.0 (0.8) | 0.124 |
| **Infective endocarditis, n (%)** | 0 (0.0) | 1 (0.3) | 0.079 | 0.0 (0.0) | 1.0 (0.3) | 0.072 |
| **Atrial fibrillation, n (%)** | 56 (80.0) | 235 (72.8) | 0.171 | 304.2 (70.8) | 292.0 (74.6) | 0.086 |
| **Coronary heart disease, n (%)** | 2 (2.9) | 1 (0.3) | 0.205 | 2.2 (0.5) | 1.2 (0.3) | 0.032 |
| **NYHA class, n (%)** |  |  | 0.347 |  |  | 0.317 |
| **I** | 1 (1.4) | 9 (2.8) |  | 41.6 (9.7) | 10.2 (2.6) |  |
| **II** | 35 (50.0) | 202 (62.5) |  | 253.5 (59.0) | 234.6 (59.9) |  |
| **III** | 33 (47.1) | 101 (31.3) |  | 128.2 (29.8) | 134.8 (34.4) |  |
| **IV** | 1 (1.4) | 11 (3.4) |  | 6.6 (1.5) | 12.0 (3.1) |  |
| **Liver disease, n (%)** | 0 (0.0) | 1 (0.3) | 0.079 | 0.0 (0.0) | 1.0 (0.3) | 0.072 |
| **Previous PCI, n (%)** | 1 (1.4) | 0 (0.0) | 0.170 | 1.0 (0.2) | 0.0 (0.0) | 0.068 |

*Note: BMI, body mass index; COPD, chronic obstructive pulmonary disease; PVD, peripheral vascular disease; NYHA, New York Heart Association; PCI, Percutaneous Coronary Intervention; SMD, standardized mean difference*

**Table S4 Baseline information after inverse-probability-weighted among different age groups in the AVR cohort.**

|  | **Aortic-Valve Replacement IPW**  **(50-59 yrs)** | | | **Aortic-Valve Replacement IPW**  **(60-70 yrs)** | | | |
| --- | --- | --- | --- | --- | --- | --- | --- |
|  | **Bioprosthetic (n=261.3)** | **Mechanical (n=268.2)** | **SMD** | **Bioprosthetic  (n=181.8)** | **Mechanical (n=185.1)** | | **SMD** |
| **Age,yrs (mean (SD))** | 54.18 (3.2) | 54.67 (2.8) | 0.164 | 64.85 (3.0) | 64.73 (3.2) | 0.039 | |
| **Female, n (%)** | 51.6 (19.8) | 81.1 (30.2) | 0.244 | 78.7 (43.3) | 81.2 (43.9) | 0.012 | |
| **BMI (mean (SD))** | 24.81 (3.3) | 24.62 (3.2) | 0.059 | 24.05 (3.4) | 24.23 (3.3) | 0.052 | |
| **Hypertension, n (%)** | 60.7 (23.2) | 67.9 (25.3) | 0.049 | 69.1 (38.0) | 70.7 (38.2) | 0.004 | |
| **Hyperlipidemia, n (%)** | 0.0 (0.0) | 5.0 (1.9) | 0.195 | 0.0 (0.0) | 4.0 (2.2) | 0.210 | |
| **Diabetes, n (%)** | 5.3 (2.0) | 11.6 (4.3) | 0.131 | 13.0 (7.1) | 8.9 (4.8) | 0.099 | |
| **stroke, n (%)** | 0.0 (0.0) | 3.0 (1.1) | 0.150 | 3.1 (1.7) | 2.9 (1.6) | 0.008 | |
| **COPD, n (%)** | 10.5 (4.0) | 11.8 (4.4) | 0.018 | 12.9 (7.1) | 13.8 (7.5) | 0.015 | |
| **PVD, n (%)** | 1.0 (0.4) | 0.0 (0.0) | 0.088 | 0.0 (0.0) | 0.0 (0.0) | | <0.001 |
| **Infective endocarditis, n (%)** | 3.0 (1.1) | 6.8 (2.5) | 0.105 | 1.0 (0.6) | 0.0 (0.0) | | 0.105 |
| **Atrial fibrillation, n (%)** | 3.1 (1.2) | 15.8 (5.9) | 0.257 | 26.3 (14.5) | 27.5 (14.9) | | 0.011 |
| **Coronary heart disease, n (%)** | 1.0 (0.4) | 6.0 (2.2) | 0.164 | 8.3 (4.5) | 6.8 (3.6) | | 0.045 |
| **NYHA class, n (%)** |  |  | 0.348 |  |  | | 0.073 |
| **I** | 26.1 (10.0) | 14.2 (5.3) |  | 4.5 (2.5) | 6.6 (3.5) | |  |
| **II** | 188.8 (72.2) | 178.9 (66.7) |  | 114.6 (63.1) | 112.5 (60.8) | |  |
| **III** | 46.5 (17.8) | 67.2 (25.0) |  | 56.4 (31.0) | 59.8 (32.3) | |  |
| **IV** | 0.0 (0.0) | 8.0 (3.0) |  | 6.3 (3.5) | 6.2 (3.4) | |  |
| **Liver disease, n (%)** | 0.0 (0.0) | 1.0 (0.4) | 0.087 | 0.0 (0.0) | 0.0 (0.0) | | <0.001 |
| **Previous PCI, n (%)** | 1.0 (0.4) | 1.0 (0.4) | 0.002 | 1.0 (0.6) | 0.0 (0.0) | | 0.105 |

*Note: BMI, body mass index; COPD, chronic obstructive pulmonary disease; PVD, peripheral vascular disease; NYHA, New York Heart Association; PCI, Percutaneous Coronary Intervention; SMD, standardized mean difference*


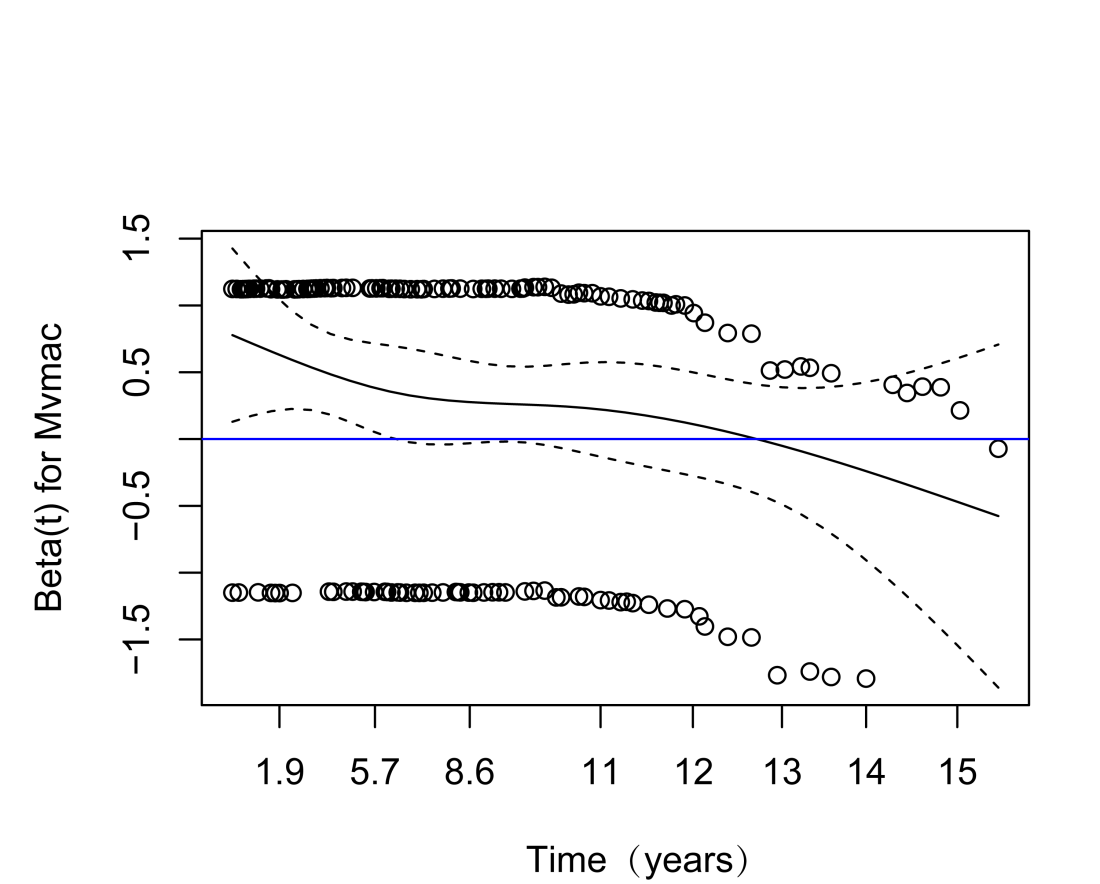


**Figure S1: Relationship between effect of valve types and time in MVR cohort.**


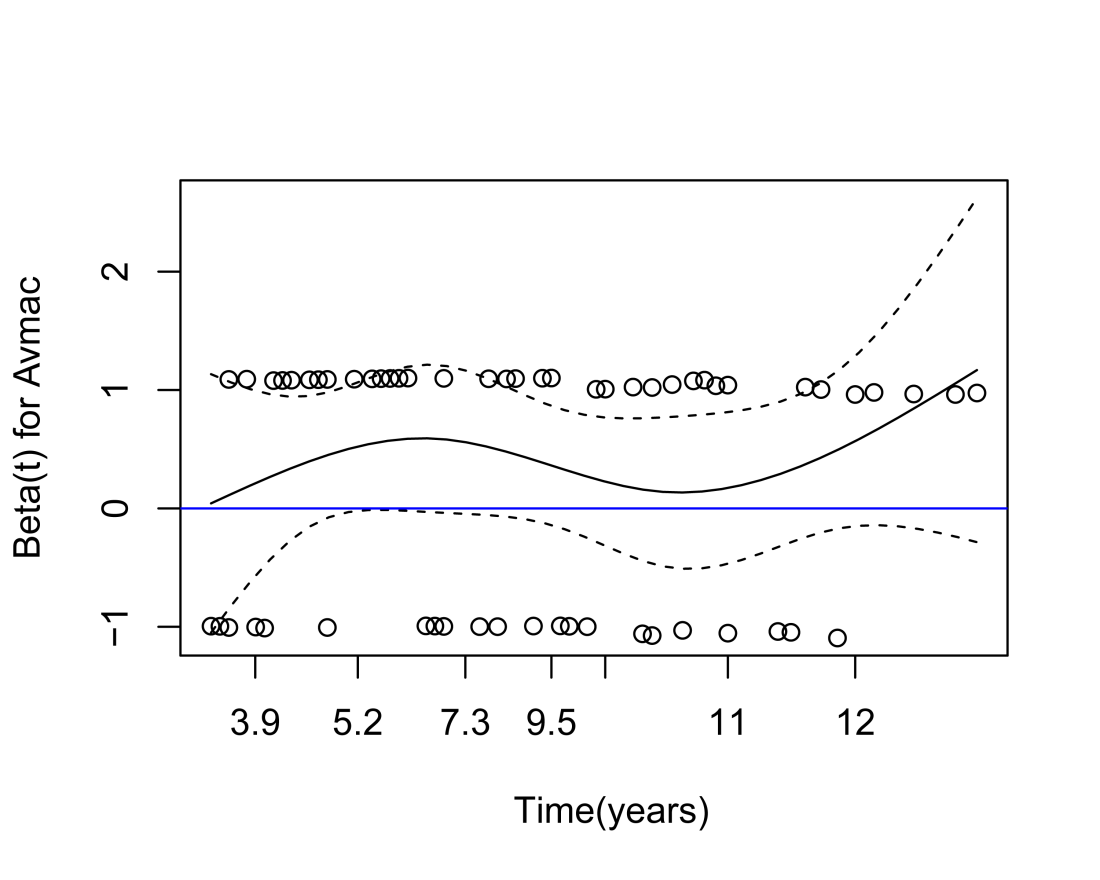


**Figure S2: Relationship between effect of valve types and time in AVR cohort.**


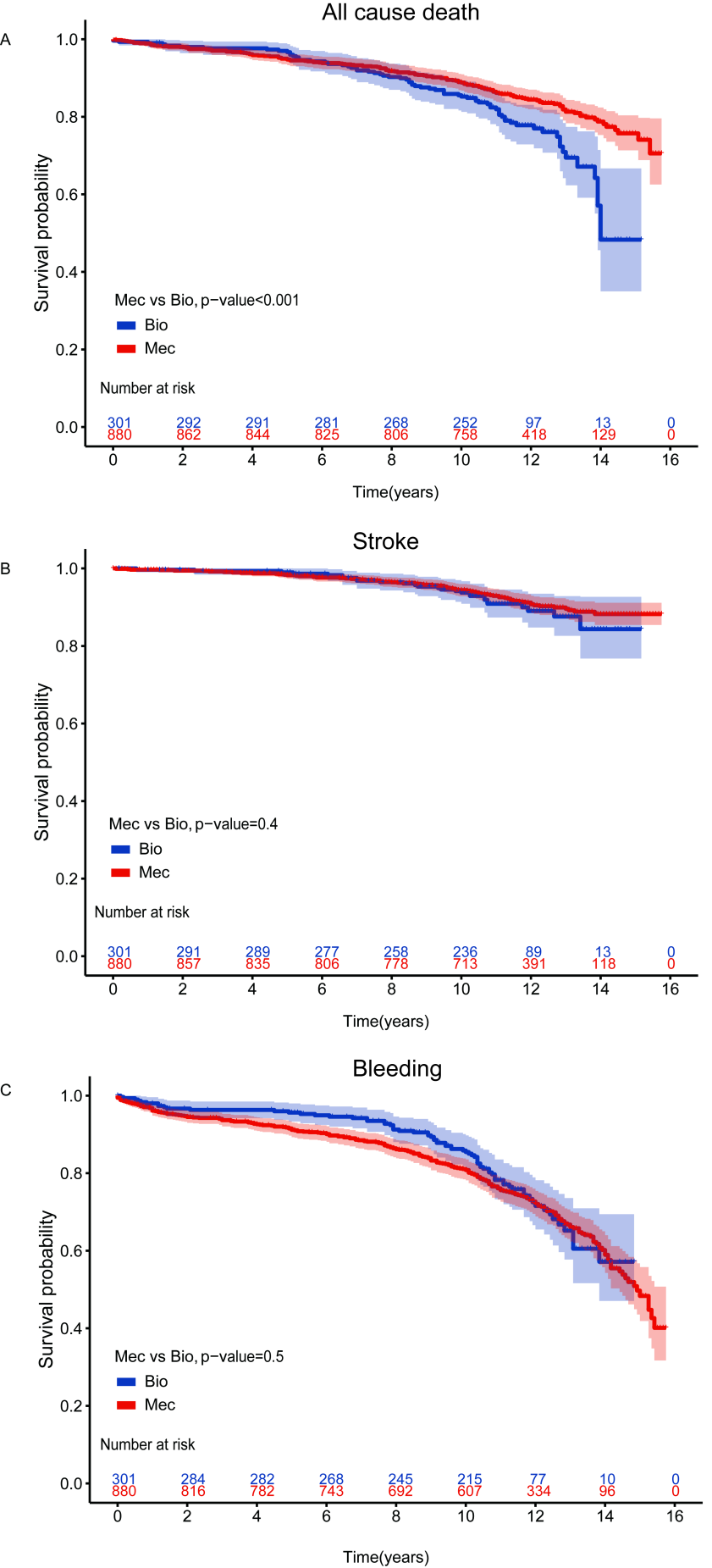


**Figure S3: Unadjusted Kaplan-Meier curve of survival among patients aged 50 – 70 years who had undergone MVR** (A) all cause death (B) Stroke events (C) Bleeding events


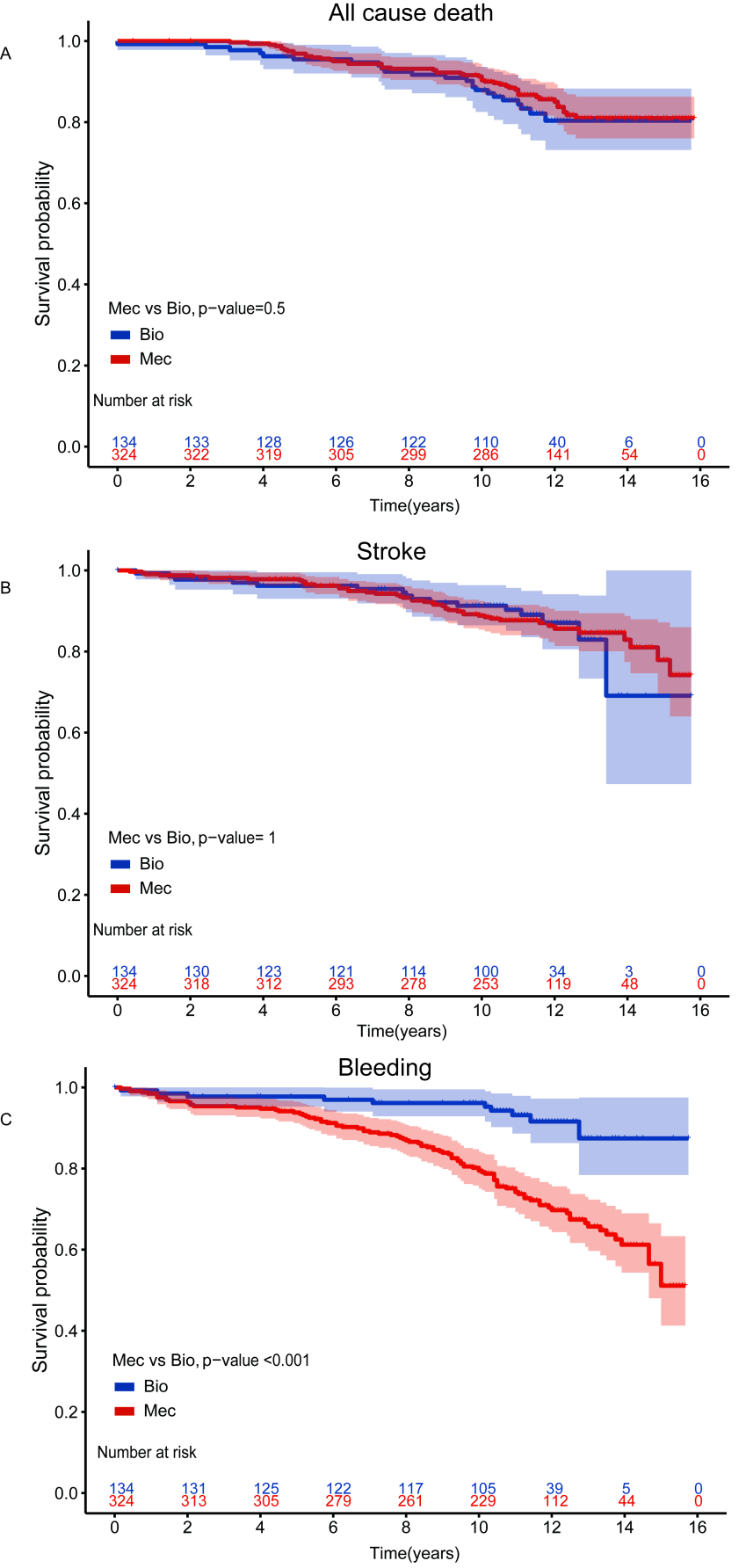


**Figure S4: Unadjusted Kaplan-Meier curve of survival among patients aged 50 – 70 years who had undergone AVR** (A) all cause death (B) Stroke events (C) Bleeding events


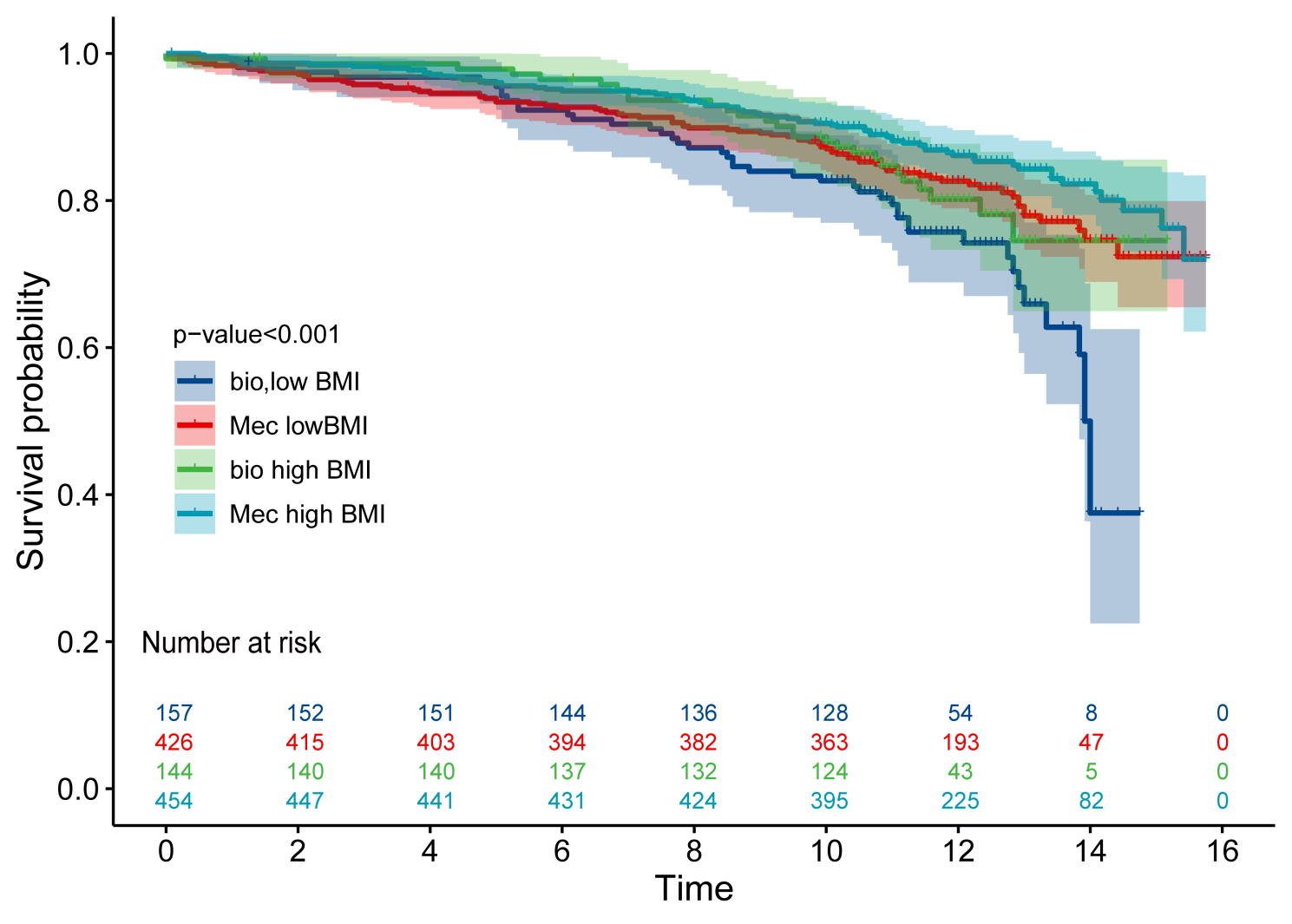


**Figure S5: Unadjusted Kaplan-Meier curve of survival among patients aged 50 – 70 years stratified by BMI and valve types in MVR cohort.**
